# Age-Specific Malaria Mortality Rates in the KEMRI/CDC Health and Demographic Surveillance System in Western Kenya, 2003–2010

**DOI:** 10.1371/journal.pone.0106197

**Published:** 2014-09-02

**Authors:** Meghna Desai, Ann M. Buff, Sammy Khagayi, Peter Byass, Nyaguara Amek, Annemieke van Eijk, Laurence Slutsker, John Vulule, Frank O. Odhiambo, Penelope A. Phillips-Howard, Kimberly A. Lindblade, Kayla F. Laserson, Mary J. Hamel

**Affiliations:** 1 Kenya Medical Research Institute/Centers for Disease Control and Prevention (KEMRI/CDC) Research and Public Health Collaboration, Kisumu, Kenya; 2 Division of Parasitic Diseases and Malaria (DPDM), Center for Global Health (CGH), CDC, Atlanta, Georgia, United States of America; 3 President's Malaria Initiative – Kenya, DPDM, CGH, CDC, Nairobi, Kenya; 4 KEMRI/CDC Research and Public Health Collaboration and KEMRI Center for Global Health Research, Kisumu, Kenya; 5 WHO Collaborating Centre for Verbal Autopsy, Umeå Centre for Global Health Research, Umeå, Sweden; 6 Liverpool School of Tropical Medicine, Liverpool, United Kingdom; 7 KEMRI Center for Global Health Research, Kisumu, Kenya; 8 Department of Clinical Sciences, Liverpool School of Tropical Medicine, Liverpool, United Kingdom; 9 Center for Global Health, CDC, Atlanta, Georgia, United States of America; Tulane University School of Public Health and Tropical Medicine, United States of America

## Abstract

Recent global malaria burden modeling efforts have produced significantly different estimates, particularly in adult malaria mortality. To measure malaria control progress, accurate malaria burden estimates across age groups are necessary. We determined age-specific malaria mortality rates in western Kenya to compare with recent global estimates. We collected data from 148,000 persons in a health and demographic surveillance system from 2003–2010. Standardized verbal autopsies were conducted for all deaths; probable cause of death was assigned using the InterVA-4 model. Annual malaria mortality rates per 1,000 person-years were generated by age group. Trends were analyzed using Poisson regression. From 2003–2010, in children <5 years the malaria mortality rate decreased from 13.2 to 3.7 per 1,000 person-years; the declines were greatest in the first three years of life. In children 5–14 years, the malaria mortality rate remained stable at 0.5 per 1,000 person-years. In persons ≥15 years, the malaria mortality rate decreased from 1.5 to 0.4 per 1,000 person-years. The malaria mortality rates in young children and persons aged ≥15 years decreased dramatically from 2003–2010 in western Kenya, but rates in older children have not declined. Sharp declines in some age groups likely reflect the national scale up of malaria control interventions and rapid expansion of HIV prevention services. These data highlight the importance of age-specific malaria mortality ascertainment and support current strategies to include all age groups in malaria control interventions.

## Introduction

During the past decade, malaria control interventions have been scaled up in Kenya and across sub-Saharan Africa. Accurate malaria burden estimates across time and age groups are necessary to measure progress and to focus programmatic interventions. Recent malaria burden estimates using different modeling approaches have led to different and controversial results, particularly in adults [1]–[5]. Murray and colleagues' findings, based in part on verbal autopsy estimates, suggest that the global proportion of malaria deaths in adults is almost always more than 40%, which is much higher than previous estimates of 10% [2], [3], [5].

Since 2001, the Kenya Medical Research Institute (KEMRI) and the U.S. Centers for Disease Control and Prevention (CDC) have monitored malaria mortality as part of the KEMRI/CDC Health and Demographic Surveillance System (HDSS) in rural western Kenya, as described in detail elsewhere [6]. The KEMRI/CDC HDSS is located in the malaria-endemic lake region of western Kenya. The region is characterized by high *Plasmodium falciparum* parasite prevalence (i.e., 38% in children <15 years in 2010) and sustained high HIV prevalence (i.e., 15% in persons >15 years from 2007–2012) [7]–[9]. The region has relatively high household insecticide-treated net ownership (i.e., 60%) but lower use estimates (i.e., 38% overall and 48% for children <5 years of age) [7]. The KEMRI/CDC HDSS has higher household insecticide-treated net ownership at 81% and usage of 57% overall and 65% for children <5 years of age [KEMRI/CDC, unpublished data]. We analyzed KEMRI/CDC HDSS data to determine the changes in malaria-specific mortality rates (MMR) and the proportion of malaria deaths across age groups from 2003–2010 and to compare these data with recent global estimates.

## Methods

### Ethics Statement

The protocol for the KEMRI/CDC HDSS was approved by the institutional review boards of KEMRI (# 1801, Nairobi, Kenya) and CDC (# 3308, Atlanta, GA). Following cultural customs, the compound head provided informed written consent for all compound members, including children, to participate in KEMRI/CDC HDSS activities. Any individual could refuse to participate at any time by verbal request. The KEMRI/CDC HDSS protocol and consent procedures, including surveillance and verbal autopsy, are approved by both KEMRI and CDC institutional review boards annually.

Data were obtained from two contiguous areas of the KEMRI/CDC HDSS, Asembo and Gem districts in Siaya County, from 2003–2010 [6]. The combined population of the areas was 147,988; 16% were children <5 years of age [10]. Malaria transmission is perennially high with peaks in May–July and October–November coinciding with the end of seasonal rains. All residences in the KEMRI/CDC HDSS were visited every four months by program staff; detailed methods previously have been reported [6], [11]. Deaths were captured through two systems: during home surveillance visits by program staff every four months and through continuous reporting by community interviewers, with ID linkage to prevent double reporting. Following a one-month grieving period, standardized verbal autopsy questionnaires were administered [6].

Verbal autopsy data were interpreted using the InterVA model version 4.RC1 (InterVA-4) [12]. InterVA-4 is a probabilistic mathematical model based on Bayesian theory used to determine cause-specific mortality fractions from standardized verbal autopsy findings [12], [13]. The InterVA-4 model allows for input of epidemiological parameters for both malaria and HIV/AIDS. In the KEMRI/CDC HDSS, both malaria and HIV/AIDS account for more than 1% of all deaths in the population and are considered “high” prevalence in the model. Conceptually, the model interprets deaths similar to the way a physician would, knowing that (s)he is working in a population with high malaria and HIV/AIDS prevalence [12].

Annual MMR per 1,000 person-years (PY) were generated, and the analyses were categorized by three age groups: children <5 years, children 5–14 years, and persons ≥15 years. The latter two age groups were collapsed into a fouth category, persons ≥5 years of age, for comparison with other estimates using only two age categories. Rates were also disaggregated by year for children <15 years. Changes in malaria mortality from 2003–2010 were analyzed by Poisson regression linear test for trend; 95% confidence intervals were calculated around rates using the PEPI (i.e., acronym for Programs for EPIdemiologists) utility for crude rates [14]. Analysis was performed using SAS version 9.1 (SAS Institute, Cary, NC) and Stata 12 (StataCorp, College Station, TX).

## Results

The total number of deaths due to malaria in the population of the KEMRI/CDC HDSS decreased from 421 per year in 2003 to 140 per year in 2010, a three-fold reduction (Table 1). During the same period, the overall MMR decreased from 3.1 to 1.0 per 1,000 PY (p<0.0001). In children <5 years, the MMR decreased from 13.2 to 3.7 per 1,000 PY, a mean reduction of 20% annually from 2003 to 2010 (p<0.0001).

**Table 1:** Malaria mortality by age group.

Year	Children <5 years				Children 5–14 years				Persons ≥15 years				Persons ≥5 years				Total Population			
	No. of malaria deaths	Rate per 1000 PYs^a^	95% Confidence Interval		No. of malaria deaths	Rate per 1000 PYs	95% Confidence Interval		No. of malaria deaths	Rate per 1000 PYs	95% Confidence Interval		No. of malaria deaths	Rate per 1000 PYs	95% Confidence Interval		No. of malaria deaths	Rate per 1000 PYs	95% Confidence Interval	
2003	294	13.2	11.7	14.7	18	0.5	0.3	0.7	109	1.5	1.2	1.8	127	1.1	0.9	1.3	421	3.1	2.8	3.4
2004	351	15.8	14.3	17.5	28	0.7	0.5	1.0	132	1.8	1.5	2.1	160	1.4	1.2	1.6	511	3.7	3.4	4.1
2005	243	11.1	9.8	12.5	22	0.6	0.4	0.9	90	1.2	1.0	1.5	112	1.0	0.8	1.2	355	2.6	2.3	2.9
2006	192	8.9	7.5	9.9	22	0.6	0.4	0.9	100	1.3	1.0	1.6	122	1.1	0.9	1.3	314	2.3	2.0	2.5
2007	109	4.7	3.9	5.7	14	0.4	0.2	0.6	53	0.7	0.5	0.9	67	0.6	0.5	0.7	176	1.3	1.1	1.5
2008	138	5.6	4.8	6.6	8	0.2	0.1	0.4	31	0.4	0.3	0.5	39	0.3	0.2	0.4	177	1.2	1.0	1.4
2009	87	3.6	3.0	4.5	17	0.4	0.3	0.7	35	0.4	0.3	0.6	52	0.4	0.3	0.6	139	1.0	0.8	1.1
2010	86	3.7	3.0	4.5	20	0.5	0.3	0.7	34	0.4	0.3	0.6	54	0.4	0.3	0.6	140	1.0	0.8	1.1

a PYs  =  Person years.

**Figure 1:**
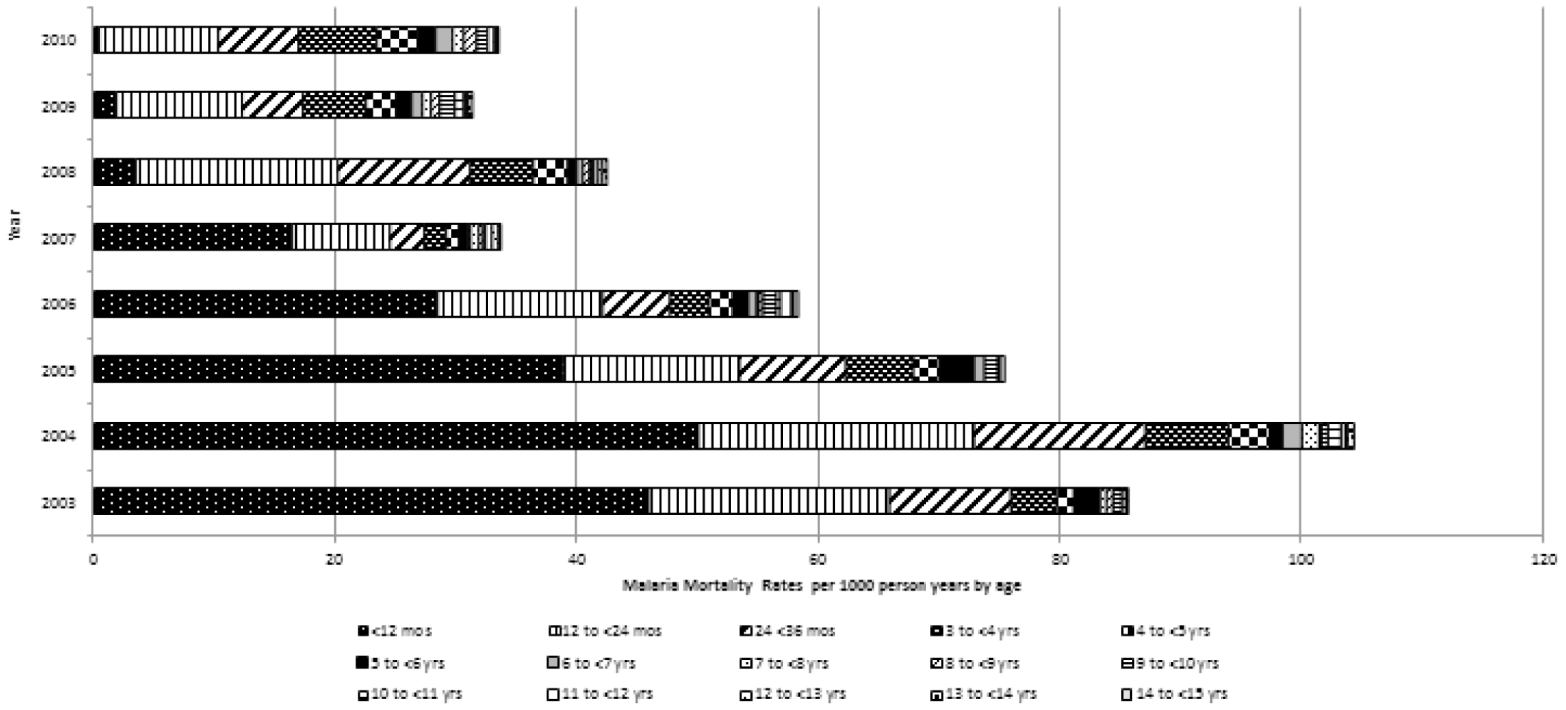
Malaria mortality rates per 1,000 person-years by age and study year. Note: x-axis indicates stacked (not aggregate) rates.

The MMR declined sharply among infants, aged 0 to <12 months, from 45.8 to 0.4 per 1000 PY (p<0.0001). Declines were also evident for children during their second year of life (i.e., aged 12 months to <24 months) decreasing from 19.9 to 10.0 (p<0.0001) and third year of life (i.e., aged 24 months to <36 months) decreasing from 10.1 to 6.6 per 1000 PY (p = 0.0002). The MMR increased among children aged 3 to 5 years from 2003–2010, although the change in rates were not statistically significant (Figure 1).

In children 5–14 years, the MMR remained relatively stable over the study period at 0.5 per 1,000 PY, with rates in 2010 mirroring rates in 2003. In this age group, annual rates by age in years fluctuated with no significant trends observed. In persons ≥15 years, the MMR decreased from 1.5 to 0.4 per 1,000 PY for a mean reduction of 20% annually (p<0.0001). When divided into only two age categories, the MMR for persons ≥5 years decreased from 1.1 to 0.4 per 1,000 PY for a mean reduction of 17% annually (p<0.0001).

**Figure 2:**
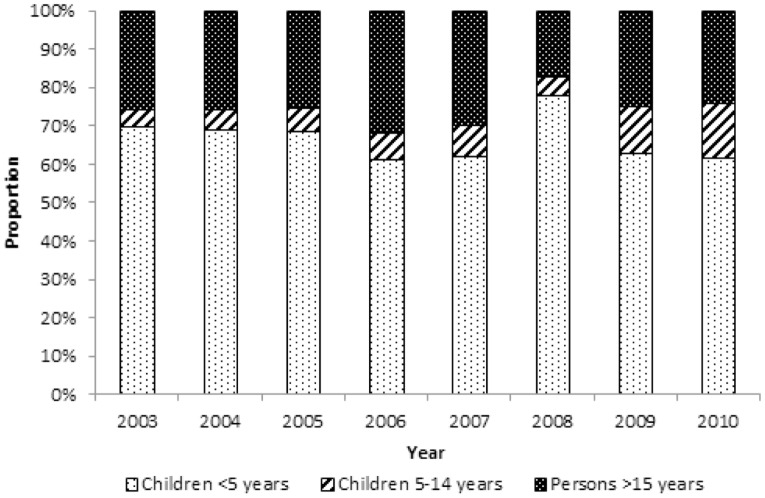
Trend in the proportion of malaria mortality by age group.

**Figure 3:**
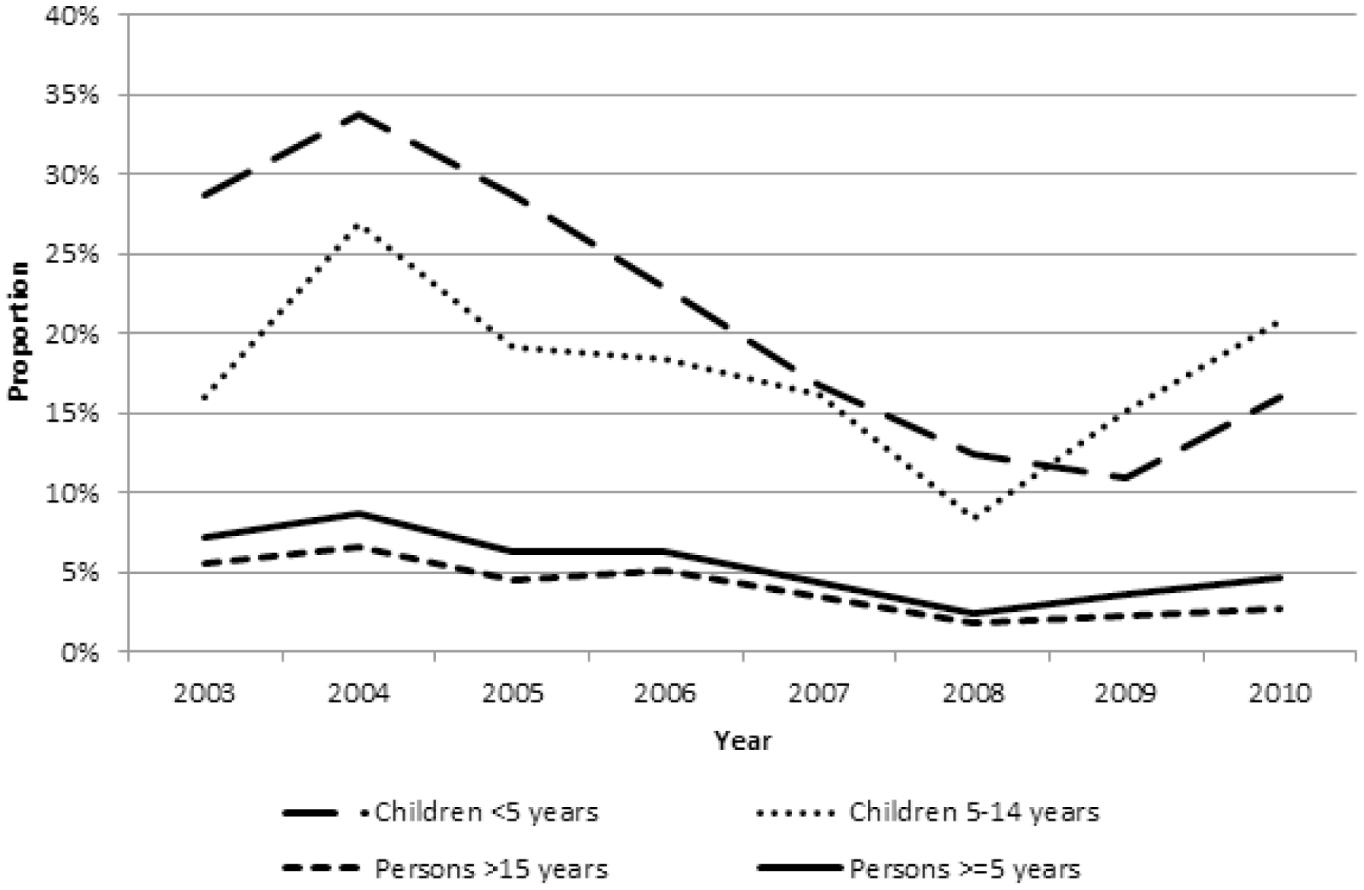
Malaria mortality as a proportion of all-cause mortality by age group.

From 2003 to 2010, the proportion of all malaria deaths by age group decreased in children <5 years from 70% to 61%, increased in children 5–14 years from 4% to 14%, and decreased slightly in persons ≥15 years from 26% to 24% (Figure 2). When divided into two age categories, the proportion of all malaria deaths in persons ≥5 years increased from 30% in 2003 to 39% in 2010. From 2003 to 2010, the proportion of malaria deaths among all deaths decreased in children <5 years from 29% to 16%, increased in children 5–14 years from 16% to 21%, and decreased in individuals ≥15 years from 6% to 3% (Figure 3). When divided into two age categories, the proportion of malaria deaths in persons ≥5 years decreased from 9% to 5%.

## Discussion

Data from the KEMRI/CDC HDSS demonstrate the substantial progress made since 2003 in reducing malaria mortality in western Kenya. The 2004 peak of malaria mortality in western Kenya coincides with the global malaria mortality trends described in the 2011 World Malaria Report and Murray and colleagues' estimates for sub-Saharan Africa [3], [5]. The accelerating trend of declining malaria mortality among children <5 years since 2003 is also consistent with the declining trend in all-cause mortality among children <5 years in sub-Saharan Africa since 2000 [15]. The MMRs in young children and persons ≥15 years have decreased dramatically from 2003 to 2010.

The MMR in children aged 5–14 years has not declined concurrently, and malaria as a proportion of all-cause mortality increased over the study period in this age group. Because the absolute number of deaths and the MMR remained relatively unchanged across the study period, the increase is probably not due to delayed acquisition of immunity. The lack of a concomitant decline in MMR in this age group might be attributed to older children not having been targeted for malaria programmatic interventions in most countries, including Kenya. Data from the KEMRI/CDC HDSS, for example, indicate that insecticide-treated net use was lowest in the 5–14 year-old age group (43% usage), and the 58% *P. falciparum* parasite prevalence in this age group was higher than all other age groups [KEMRI/CDC, unpublished data].

These data provide insight into a shift of mortality burden from infants to slightly older children who, as discussed above, do not seem to have benefited as much as younger children from malaria intervention. Continued mortality surveillance is needed to ascertain if deaths in older children proportionately increase, or increase in absolute terms, as malaria transmission is reduced towards pre-elimination levels, as suggested in the literature [16], [17]. In order to reach global malaria control and elimination goals, interventions will have to be scaled up in age groups that historically have been excluded and surveillance strengthened across all age groups to monitor impact [18].

The considerable decline in MMRs in the KEMRI/CDC HDSS likely reflects, in part [19], the growth of malaria and HIV interventions in Kenya over the study period. For malaria, the introduction and scale up of key interventions recommended by Roll Back Malaria occurred during the study period [20]. In 2004, Kenya changed the national malaria treatment policy to recommend artemisinin-based combination therapy (ACT) as the first-line treatment for uncomplicated malaria, and by 2006, the new ACT guidelines were implemented countrywide [21], [22]. However, by 2010, only 18% of febrile children <5 years were treated with an ACT for uncomplicated malaria, suggesting that most malaria cases were being treated with ineffective medications or monotherapies throughout the study period [7]. Kenya did not adopt national policy to introduce malaria rapid diagnostic tests (RDTs) in endemic areas until 2012. Therefore, the majority of treatment for malaria during the study period was likely based on clinical diagnosis. The national malaria control program targeted 3.4 million children <5 years during the first mass insecticide-treated net campaign in 2006 and conducted a second mass net campaign in 2010–2011 to provide universal coverage in malaria endemic and epidemic-prone areas. The mass campaigns increased net use by children <5 years from 4.6% in 2003 to 42.2% in 2010 [7], [23]. Since 1998, the national malaria control program has recommended intermittent preventive therapy for pregnant women, and by 2010, 25% of women received two or more doses of sulphadoxine-pyrimethamine during pregnancy compared to 13% in 2007 [7].

Additionally, the expansion of HIV prevention, treatment and care services are likely to have significantly contributed to the gains achieved in mortality reduction, particularly among the youngest children. Before 2003, most Kenyans had no access to HIV services, but by 2008, 1 in 5 HIV-positive adults were receiving HIV care, with half of those eligible receiving antiretroviral therapy [24]. A South African study found that for every 100 HIV-infected persons on antiretroviral therapy, 6.3 fewer deaths occurred the following year, including among children [25]. Studies in the KEMRI/CDC HDSS showed a halving of all-cause and HIV-specific mortality among young adult females from 2003 to 2009 [26]. We separately evaluated trends in HIV-specific mortality rates using the InterVA-4 model; among adults ≥ 15 years, HIV-specific mortality declined significantly over the study period (p<0.0001), but among children <15 years, HIV-specific mortality by year of age did not show similar trends. Increased uptake of services to prevent mother-to-child HIV transmission was likely to have contributed to the particularly dramatic decrease in malaria-specific mortality among children <36 months potentially through two mechanisms. First, HIV deaths in infants and young children were likely to have been misclassified as malaria deaths because malaria was both common and more culturally acceptable. Second, preventing HIV infection led to fewer infants and young children with severe immunodeficiencies and the associated higher risk of death from malaria and HIV co-infection. Therefore, both malaria and HIV prevention and treatment interventions are likely to have contributed to the dramatic reductions in MMR in children <5 years and adults ≥15 years.

Malaria mortality as a proportion of total mortality decreased substantially from 2003 to 2010 in children <5 years and persons ≥15 years. In 2008 among children <5 years, the proportion of deaths due to malaria was 12% in the KEMRI/CDC HDSS, which is lower than both the 2008 estimates of 24% by Murray and 16% by Black for child deaths due to malaria in Africa [5], [27]. Recognizing the limitations of verbal autopsy for assigning cause of death, we also examined all deaths with the symptom of acute febrile illness (AFI) before death (Figure S1). Between a third to a half of all deaths in children <5 years and 5–14 years had an acute febrile illness, which remained relatively constant over the study period. Similar to the declines observed in MMR, between 2003 and 2010, overall AFI mortality rate also decreased from 5.4 to 2.1 per 1,000 PY (p<0.001): 23.9 to 9.7 per 1,000 PY (p<0.001) in <5 years; 0.8 to 0.7 (p = 0.08) in 5–14 years; and 2.8 to 0.9 (p<0.001) in ≥15 years. There was a substantial decline in deaths in children below 3 years of age. Concurrently, as the number of deaths with AFI declined, the proportion of deaths ascribed to malaria in those with AFI rose over time across all age groups (Table S1). Together, this provides some assurance that malaria as a cause of death was not being under-estimated over time. Among persons ≥15 years in the KEMRI/CDC HDSS in 2010, only 3% of deaths were due to malaria. This percentage is consistent with recent findings from Byass and colleagues that examined the distribution of cause-specific mortality across five African countries using InterVA-4 methodology [28].

The lowest malaria mortality as a proportion of all-cause mortality occurred in 2008 for persons >5 years and in 2009 for children <5 years. The subsequent increase in proportional malaria mortality in 2009–2010 might have been a result of internal population displacements due to post-election civil conflict and wide-scale public sector stock outs of ACTs [11]. Disruption of health services and population displacement resulting from post-election violence were identified as factors causing an upsurge in HIV morbidity and mortality in the KEMRI/CDC HDSS in 2008 [29]. However, the causes of the increase in proportional malaria mortality might be multifactorial and include natural variation in cyclical, seasonal, and annual malaria transmission patterns, linked to weather patterns.

Among all malaria deaths, the proportion that occurred in individuals <15 years remained relatively stable at ∼75%. We note the proportion of non-child malaria deaths (∼25%) is higher than the WHO global estimate of 10% and comparable to estimates for areas with high malaria transmission in Africa by Murray and colleagues [2], [5]. Misclassification bias resulting in the overestimation of malaria deaths due to HIV-associated mortality in the early years of the study when HIV-associated mortality peaked is a potential study limitation [22]. A recent analysis of cause-specific mortality ratios between HIV-negative and HIV-positive individuals in five African countries showed that there was a lower malaria-specific mortality ratio among HIV-positive individuals than the all-cause mortality ratio associated with HIV infection [15]. Because cause of death was assigned consistently over the study period using the InterVA-4 model, any misclassification bias would be consistent over the study period and not likely to alter the direction of the change in MMRs. However, misclassification errors occur in all forms of cause-of-death assignment, including verbal autopsy with InterVA-4 modeling, and should be recognized in any study relying on cause-specific mortality data. In particular, misclassification bias that may differ across ages requires careful validation before the proportion of age-specific malaria deaths can be reliably estimated using VA methods.

## Conclusions

Substantial gains have been made in malaria prevention and control in Kenya over the last decade. Data from this study demonstrates a lower proportion of malaria deaths in children <5 years compared to other estimates from Africa. For persons ≥15 years, proportional malaria mortality in 2010 is comparable to recent estimates for high malaria transmission countries in Africa. Malaria remains a major public health concern for all age groups, even in areas of relatively high insecticide-treated net ownership and use. These data provide some assurance of the potential contribution current malaria control strategies, such as Roll Back Malaria strategy of universal coverage with insecticide-treated nets, may accord and support strategies to reach older children and adults with malaria prevention and control interventions. Development and implementation of new malaria surveillance and control tools to ascertain population groups at risk, including by age, are necessary for continued progress towards global goals for malaria control and elimination.

## Supporting Information

Figure S1 **Acute febrile illness mortality rates per 1,000 person-years by age and study year.** Note: x-axis indicates stacked (not aggregate) rates.
(EPS)

Table S1 **Proportion of malaria deaths out of those with acute fever, by age and year.**
(DOCX)
