# Supplementary material for: Age-Specific Malaria Mortality Rates in the KEMRI/CDC Health and Demographic Surveillance System in Western Kenya, 2003–2010
Source: PLoS One. 2014 Sep 2;9(9):e106197. doi: 10.1371/journal.pone.0106197 (PMC4152016; doi:10.1371/journal.pone.0106197)
Supplement: Table S1 — Proportion of malaria deaths out of those with acute fever, by age and year. (DOCX) [file pone.0106197.s002.docx]

Table S1: Proportion of malaria deaths out of those with acute fever, by age and year

|  | | | | | | | | |
| --- | --- | --- | --- | --- | --- | --- | --- | --- |
|  | 2003 | 2004 | 2005 | 2006 | 2007 | 2008 | 2009 | 2010 |
| <12 m | 0.22 | 0.29 | 0.30 | 0.28 | 0.27 | 0.41 | 0.44 | 0.54 |
| 12-<24 m | 0.51 | 0.57 | 0.49 | 0.45 | 0.41 | 0.64 | 0.76 | 0.69 |
| 24-<36 m | 0.61 | 0.61 | 0.58 | 0.35 | 0.38 | 0.63 | 0.72 | 0.79 |
| 3- <4 yrs | 0.45 | 0.8 | 0.67 | 0.5 | 0.42 | 0.74 | 0.81 | 0.72 |
| 4- <5 yrs | 0.45 | 0.61 | 0.37 | 0.53 | 0.43 | 0.68 | 0.78 | 0.8 |
| 5- <6 yrs | 0.58 | 0.56 | 0.9 | 0.33 | 0.5 | 0.4 | 0.82 | 0.75 |
| 6- <7 yrs | 0.33 | 0.67 | 0.25 | 0.5 | 0.33 | 0.6 | 0.5 | 0.67 |
| 7- <8 yrs | 0.11 | 0.63 | 0 | 0 | 0.6 | 0.2 | 0.6 | 0.63 |
| 8- <9 yrs | 0.33 | 0.25 | 0 | 0 | 0 | 0.5 | 0.75 | 0.8 |
| 9-<10 yrs | 0.5 | 0.67 | 0.67 | 0.5 | 0 | 1.0 | 0.8 | 0.83 |
| 10-<11 yrs | 1.0 | 0.75 | 0 | 0.4 | 1.0 | 0.5 | 1.0 | 1.0 |
| 11-<12 yrs | 0 | 0.5 | 0 | 0.5 | 0.25 | 0.67 | 0 | 1.0 |
| 12-<13 yrs | 0.25 | 0.5 | 0 | 0 | 0.67 | 0.67 | 0 | 0 |
| 13-<14 yrs | 0 | 0 | 0 | 1.0 | 0.5 | 0 | 1.0 | 1.0 |
| 14-<15 yrs | 0 | 0 | 0.5 | 0 | 0 | 0 | 0 | 0 |
| ≥15 yrs | 0.16 | 0.18 | 0.16 | 0.16 | 0.12 | 0.19 | 0.31 | 0.23 |
